# Supplementary material for: Development of Policy-Relevant Indicators for Injury Prevention in British Columbia by the Key Decision-Makers
Source: Int J Environ Res Public Health. 2021 Nov 11;18(22):11837. doi: 10.3390/ijerph182211837 (PMC8621597; doi:10.3390/ijerph182211837)
Supplement: Supplementary file 1 [file ijerph-18-11837-s001.zip › ijerph-1373623-supplementary.pdf]

**Supplementary Table S1.** Indicator Specification Template.

| **Indicator name** |
| --- |
| Variation and Disaggregation of Indicator |
| Definition of relevant terms |
| Justifications for this indicator |
| Operational definition of a case |
| Method of calculation |
| Numerator |
| Denominator |
| Data sources, availability and quality/Years represented |
| Unit of measurement |
| Scope of indicator |
| Specifications of data needed |
| Limitations |
| How to use this indicator |

**Supplementary Table S2:** Road Safety indicators after applying thresholds

| Indicator Domain | Indicators |
|---|---|
| **Safe Speed (SS)** | 1. Number of kilometres of road with Point to Point Automated Speed Enforcement.<br>2. Number of locations employing single-point Automated Speed Enforcement (i.e. photo radar or 'Speed on Green').<br>3. Number of vehicle impoundments for speeding per 100,000 populations.<br>4. Presence of Official Community Plan that incorporates the Safe System Approach speed limits<br>5. Percentage of roads in your jurisdiction in that adhere to Safe Systems Approach speed limits (30 km/hour on urban roads)<br>6. Highway speed distribution (mean and variance).<br>7. Rate of Speed Compliance on Rural Highways.<br>8. Rate of Speed Compliance on Urban Roads. |
| **Safe Road (SR)** | 1. Presence of measures to improve pedestrian safety in and around schools (e.g. a "Safe Route to School program"; 30 km/h speed limits surrounding schools, etc.).<br>2. Total length (km) of off-street pedestrian walkways.<br>3. Ratio of total length of current sidewalk (km) to current road (km) in a designated area (e.g. neighbourhood, community, municipality, etc.).<br>4. Kilometres of roads with posted speeds of 80 km/h or faster that have median barriers to prevent cross-over crashes<br>5. Kilometres of roads with posted speeds of 80 km/h or faster that have road side barriers to prevent run-off-road crashes<br>6. Total length (km) of unprotected on-road bike lanes.<br>7. Total length (km) of Multi-Use Pathways (MUPs).<br>8. Presence of designated heavy truck traffic route. |
| **Safe vehicle (SV)** | 1. Percentage of vehicles registered in BC with crash avoidance systems that include pedestrian/cyclist detection and automatic braking. |

| | |
|---|---|
| **Safe Road User (SRU)** | 1. Designated traffic officer full-time equivalent (FTE) per 100,000 populations.<br>2. Number of intersections with safety cameras<br>3. Number of signalized intersections with red light cameras<br>4. Number of intersections with red light and speed cameras<br>5. Number of signalized intersections with accessibility infrastructure for pedestrians with visual or auditory impairments (ex. audible signals)<br>6. Percentage of signalized intersections that have an exclusive left turn lane AND left turn light phase.<br>7. Number of 24 Hour Roadside prohibitions (when an officer suspects a driver is impaired, their driver's licence can be suspended for up to 24 hours) for alcohol.<br>8. Number of 24-Hour Roadside prohibitions (when an officer suspects a driver is impaired, a driver's licence can be suspended for up to 24 hours) for impairing drugs.<br>9. Number of Immediate Roadside Prohibitions (when an officer uses an Approved Screening Device to confirm that a driver is above the limit of breath/blood alcohol level, the driver's license can be suspended and the vehicle impounded)<br>10. Number of traffic violations per licensed drivers per time period (e.g. month or year) in your jurisdiction related to alcohol.<br>11. Number of traffic violations per licensed drivers per time period (e.g. month or year) in your jurisdiction related to distracted driving (e.g. cell phone use).<br>12. Number of traffic violations per licensed drivers per time period (e.g. month or year) in your jurisdiction related to high risk driving.<br>13. Number of traffic violations per licensed drivers per time period (e.g. month or year) in your jurisdiction related to drug impairment.<br>14. Recidivism rate for alcohol-impaired driving.<br>15. Percentage of drivers observed running red lights.<br>16. Percentage of drivers observed using a cell phone while driving.<br>17. Percentage of drivers observed wearing seatbelts.<br>18. Ratio of trips made using public transit compared to trips made by private vehicles.<br>19. Ratio of walking and cycling trips to private vehicle trips. |
| **Outcome (O)** | 1. Time to reach to the nearest trauma centre or an emergency from motor vehicle crash.<br>2. Number of cases with significant delay in discovery time or to post crash care.<br>3. Percentage of fatal crashes reported according to speed limit at site of crash.<br>4. Total road-related fatalities per 100,000 population<br>5. Number/percent of fatal crashes reported according to speed limit at site of crash. (Original indicator: Total km of highways and roads by speed limits and crash<br>6. Percentage of serious injury crashes reported according to speed limit at site of crash.<br>7. Total road-related serious injuries (overnight hospital admissions) visits per 100,000 population<br>8. Percentage of motor vehicle crashes caused by a vehicle crossover (i.e. a crash where a car crosses the centre line and hits oncoming traffic).<br>9. Percentage of motor vehicle crashes caused by a vehicle running off the road.<br>10. Percentage of motor vehicle crashes caused by red-light running.<br>11. Total road-related emergency department visits |

**Supplementary Table S3.** Seniors Falls Indicators after applying thresholds

| Indicator Domain | Indicators |
|---|---|
| **Health Systems (HS)** | 1. Number of fall-related injury fatalities among people aged 65 years and older<br>2. Number of fall-related injury hospitalizations among people aged 65 years and older<br>3. Rate of fall-related injury hospitalizations among people aged 65 years and older<br>4. Number of hip fracture emergency department visits in persons aged 65 years and older<br>5. Repeat ED (two or more) visits for falls in the past 12 months at the beginning of the rolling 12 month period per 100,000 seniors aged 65 years and older<br>6. Disability Adjusted Life Year (DALY) due to fall-related injury<br>7. Indirect Cost of falls-related injury<br>8. Direct Cost of falls-related injury<br>9. Waiting time for surgery following fall related injury |
| **Health service coverage (HSC)** | 1. Falls risk screening assessment coverage at primary care level<br>2. Strength & balance exercise program coverage<br>3. Home risk assessment coverage |
| **Policy (PLC)** | 1. The province has designated, qualified staff members responsible for fall prevention<br>2. Availability of has a fall prevention coalition/ network<br>3. Availability of a report on fall related morbidity and mortality<br>4. Availability of a strategic plan or framework to decrease falls and falls related injuries among community living older adults<br>5. Availability of education programs to all healthcare staff on fall related injury prevention<br>6. Availability and timely updating of a website/platform for senior fall- related injury prevention |

**Supplementary Table S4.** All-injury indicators after applying thresholds

| Indicator Domain | Indicators |
|---|---|
| **Health Systems (HS)** | 1. Number of unintentional injury fatalities<br>2. Rate of unintentional injury fatalities<br>3. Number of drug overdose fatalities<br>4. Number of unintentional injury hospitalizations<br>5. Rate of unintentional injury hospitalizations<br>6. Rate of injury-related long bone fractures<br>7. Length of hospital stay related to injury<br>8. Percentage discharged to long-term care following injury<br>9. Percentage discharged to community with home care following injury<br>10. Number of injury hospitalizations associated with alcohol<br>11. Number of injury hospitalizations associated with other Illicit drug use<br>12. Percentage of sports specific injuries<br>13. Number of (ED) visits for unintentional injuries<br>14. Number of injury ED visits associated with alcohol<br>15. Number of injury ED visits associated with other Illicit drug use<br>16. Self reported unintentional injury rate per 1000 population<br>17. Poisoning helpline utilization Potential years of life lost<br>18. Disability Adjusted Life Year (DALY)<br>19. Indirect cost of injury<br>20. Direct cost of injury<br>21. Treatment coverage for alcohol and drug dependence |
| **Additional indicators (AI)** | 1. Percentage of adult binge drinking<br>2. Proportion of adults following the low risk drinking guidelines<br>3. Number and proportion of communities with access to water safety education/programs<br>4. Number and proportion of children and youth enrolled in "learn to swim" programs in a specific year<br>5. Number of communities with by-laws requiring pool fencing<br>6. Standard for hot water tap maximum temperature<br>7. Window guard by-law<br>8. Requirement for playgrounds to meet CSA standards<br>9. Number and proportion of homes in a community with working smoke detectors, tested fire extinguishers and carbon monoxide detectors<br>10. Percentage of bicycle helmet use<br>11. Number of poisoning helpline utilization<br>12. Availability of fire and ambulance services in a community within a defined response time |

**Supplementary Table S5.** Final Indicators, grouped by variations and disaggregations, with additional disaggregations included as part of the specification process

| Indicator Name | | Major Disaggregations |
|---|---|---|
| **Road Safety** | | |
| **Access to timely and appropriate care**<br>Variations: Distance (km) of motor vehicle crash from nearest trauma centre; Number of cases with significant delay in discovery time or to post crash care | Outcome | • Health Authority<br>• Municipality<br>• Dissemination Area<br>• Census Agglomeration<br>• Metro, Urban/Rural, Rural or Remote<br>• Material Deprivation<br>• Social Deprviation<br>• Age Group<br>• Sex<br>• Year<br>• Month<br>• Day<br>• Time of Day<br>• Location<br>• Road Type<br>• Road User Type<br>• Vehicle Type<br>• Type of Crash<br>• Crash Counterpart<br>• Contributing Factor<br>• Kilometre of road travelled<br>• Speed Limit of Roadway<br>• Latitude and Longitude<br>• Highways with Variable Speed Limits |
| **Road-related fatalities**<br>Variations: Number, rate, and proportion of road-related fatalities; Number, age adjusted rates, and proportion (percentage) of road-related fatalities according to speed limit at site of crash | Outcome, Safe Speeds | |
| **Road-related serious injuries**<br>Variations: Total, rate, and proportion (percentage) of road-related serious injuries according to speed limit at site of crash; Total age-adjusted rate, and proportion of road-related serious injuries | Outcome | |
| **Road-related ED visits**<br>Variations: Total, crude rate, and proportion of road-related emergency department visits; Location/geospatial map of road-related incidents resulting in emergency department visits | Outcome | |
| **Automated Speed enforcement**<br>Variations: Number of sections of point to point, total km covered by point to point, and proportion of total highway km covered by point to point; Number of ASE installations; Number of signalized intersections with red light cameras; Number of signalized intersections with speed cameras; Number of intersections with red light and speed cameras | Safe Speeds, Safe Roads, Safe Road Users | |
| **Speed compliance**<br>Variations: Frequency distribution of speeds, proportion of drivers at each speed and range of speeds by speed limit | Safe Speeds | |
| **Signalised intersection safety**<br>Variations: Number of signalized intersections with accessibility infrastructure; Percentage of signalized intersections that have an exclusive left turn lane AND left turn light phase | Safe Roads | |
| **Designated heavy truck traffic route**<br>Variations: Total number and proportion of designated heavy truck traffic routes | Safe Roads | |
| **Pedestrian safety in school zones**<br>Variations: Number and proportion of Safe Route to School programs; Number and proportion of 30 km/h speed limits surrounding schools | Safe Roads | |

| | |
|---|---|
| **Pedestrian safety** Variations: Total km of off-street pedestrian walkways; Ratio of total length of current sidewalk (km) to current road (km) | Safe Roads |
| **Enhanced road designs** Variations: Length of km and proportion of road with median barrier; Length of km and proportion of road with side barrier | Safe Roads |
| **Passive safety technology** Variations: Number and proportion passive safety technology | Safe Vehicles |
| **Traffic enforcement** Variations: Number and rate of designated traffic officer full-time equivalent | Safe Road Users, Safe Speeds |
| **Traffic legislative initiatives to enhance traffic safety** Variations: Number, rate, and proportion of 24 Hour Roadside prohibitions for alcohol; Number, rate, and proportion of 24 Hour Roadside prohibitions for impairing drugs; Number, rates, and proportion of immediate roadside prohibitions | Safe Road Users, Safe Speeds |
| **Traffic violations** Violations: Number and rate of traffic violations per licensed driver per 5-year period related to alcohol; Number and rate of traffic violations per licensed driver per 5-year period related to distracted driving; Number and rate of traffic violations per licensed driver per 5-year period related to high-risk driving; Number and rate of traffic violations per licensed driver per 5-year period related to drug-impaired driving | Safe Road Users, Safe Speeds |
| **Safe and unsafe driving behaviours** Variations: Number, rate, and proportion of drivers observed running red lights during roadside surveys; Number and rate of drivers observed using a cell phone while driving; Number and rate of drivers observed wearing seatbelts | Safe Road Users |
| **Active transportation** Variations: Ratio of trips using public transit; Ratio of trips that were walking; Ratio of trips that were cycling | Safe Road Users |
| **Unsafe speed and traffic crashes** Variations: Proportion of vehicle crossover collisions resulting in fatalities or serious injuries; Proportion of collisions due to vehicles run off the road that resulted in fatalities or serious injuries; Proportion of collisions due to red light running that resulted in fatalities or serious injuries | Outcome, Safe Speeds |

| | | |
|---|---|---|
| **Speeding related vehicle impoundments**<br>Variations: Number and age adjusted rate of vehicle impoundments for speeding | Safe Speeds | |
| **Evidence-based speed limit approach**<br>Variations: Number and proportion of Official Community Plan that incorporates the Safe System Approach speed limits | Safe Speeds | |
| **Unprotected and protected on-road bike lanes**<br>Variations: Length and ratio of unprotected and protected on-road bike lanes | Safe Roads | |
| **Multi-use pathway**<br>Variations: Total length and proportion of multi-use pathways | Safe Roads | |
| **Road-related injury costs**<br>Variations: DALY due to road-related injury; DALY due to road -related injury for ED visits; DALY due to road -related injury for hospitalizations; DALY due to road -related injury for fatalities; Indirect costs and cost per capita due to road-related injury; Indirect costs due to road -related injury for ED visits; Indirect costs due to road -related injury for hospitalizations; Indirect costs due to road -related injury for fatalities; Direct costs and cost per capita due to road -related injury; Direct costs due to road -related injury for ED visits; Direct costs due to road -related injury for hospitalizations; Direct costs due to road -related injury for fatalities | Outcome | |
| **Seniors Falls** | | |
| **Fall-related fatalities**<br>Variations: The total, age-adjusted rate, and proportion of fall fatalities | | |
| **Fall-related injury hospitalization**<br>Variations: The total, age-adjusted rate, and proportion of fall hospitalizations; Length of hospital stay; Number and proportion of patients discharged to long-term care post fall injury; Number and proportion of patients discharged to long-term care post fall injury; Number and proportion of patients discharged to community home care following fall injury | | • Health Authority<br>• Municipality<br>• Dissemination Area<br>• Metro, Urban/Rural, Rural or Remote<br>• Material Deprivation<br>• Social Deprviation<br>• Agre Group<br>• Sex<br>• Day<br>• Time of Day<br>• Fall type<br>• Injury Type<br>• Place of Occurrence<br>• Latitude and Longitude |
| **Fall-related ED visits and repeat visits**<br>Variations: Number, rate, and proportion of hip fracture emergency department visits; Number, rate, and proportion of hip fracture emergency department visits among seniors; Number, rate, and proportion of repeat ED visits for falls in the past 12 months among seniors | | |
| **Fall-related costs**<br>Variations: DALY due to fall-related injury; DALY due to fall-related injury for ED visits; DALY due to fall-related injury for hospitalizations; DALY due to fall-related injury for fatalities; Indirect costs and cost per capita due to fall-related injury; Indirect costs due to fall-related injury for ED visits; Indirect costs due to fall-related injury for hospitalizations; Indirect costs due to fall-related injury for fatalities; Direct costs | | |

| | |
|---|---|
| and cost per capita due to fall-related injury; Direct costs due to fall-related injury for ED visits; Direct costs due to fall-related injury hospitalizations; Direct costs due to fall-related injury fatalities | |
| **Wait time for surgery**<br>Variations: Amount of fall-related surgery wait time | |
| **Health service coverage**<br>Variations: Number and proportion of falls risk screening assessment coverage at primary care level; Number and proportion of strength and balance exercise program coverage; Number and proportion of home risk assessment coverage | |
| **Fall prevention designated staff**<br>Variations: Number, rate, and proportion designated, qualified staff members responsible for fall prevention | |
| **Availability of fall prevention resources and plans**<br>Variations: Number and proportion of fall prevention coalition/networks; Number and proportion of reports on fall-related morbidity and mortality; Number of strategic plans or frameworks to decrease falls and fall-related injuries among community living older adults; Number of updated website/platforms for senior fall-related injury prevention | |
| **All-Injury** | |
| **Number and rate of unintentional injury fatalities**<br>Variations: Number, proportion, and rate of unintentional injury fatalities; Number, proportion, and rate of drug overdose fatalities; Number, proportion, and rate of transport-related injury fatalities; Number, proportion, and rate of fall-related injury fatalities; Number, proportion, and rate of unintentional poisoning fatalities; Number, proportion, and rate of suicides; Number, proportion, and rate of homicides; Number, proportion, and rate of drowning-related fatalities; Number, proportion, and rate of fire and hot substance-related injury fatalities; Number, proportion, and rate of suffocation and choking fatalities; Number, proportion, and rate of sport-related injury fatalities; Number, proportion, and rate of concussion-related injury fatalities; Number, proportion, and rate of other injury fatalities | • Health Authority<br>• Health Service Delivery Area<br>• Local Health Area<br>• Municipality<br>• Dissemination Area<br>• Metro, Urban/Rural, Rural or Remote<br>• Material Deprivation<br>• Social Deprviation<br>• Age Group<br>• Sex<br>• Day<br>• Time of Day<br>• Injury Type<br>• Mechanism of Injury<br>• Other Diagnosis<br>• Mechanism of Injury and Other Diagnosis<br>• Underlying Cause of Death<br>• Contributing Cause of Death<br>• Underlying and Contributing Cause of Death<br>• Substance Typee<br>• Latitude and Longitude |
| **Number and rate of unintentional injury hospitalizations**<br>Variations: Number, proportion, and rate of unintentional injury hospitalizations; Number, proportion, and rate of drug overdose hospitalizations; Number, proportion, and rate of transport-related injury hospitalizations; Number, proportion, and rate of fall-related injury hospitalizations; Number, proportion, and rate of unintentional poisoning hospitalizations; Number, proportion, and rate of self-harm hospitalizations; Number, proportion, and rate of assault hospitalizations; Number, proportion, and rate of drowning-related hospitalizations; Number, proportion, and rate of fire and hot substance-related injury hospitalizations; Number, proportion, and rate of suffocation and choking hospitalizations; Number, proportion, and rate of sport-related injury hospitalizations; Number, proportion, and rate of concussion-related injury hospitalizations; Number, proportion, and rate of other injury hospitalizations | |
| **ED visits for unintentional injuries**<br>Variations: Number, rate and proportion of unintentional injury ED visits; Number, rate and proportion of unintentional injury ED visits by | |

| |
|---|
| mechanism of injury; Number, rate and proportion of unintentional injury ED visits by mechanism of injury and other diagnosis; Number, rate and proportion of unintentional injury ED visits associated with illicit drugs |
| **PYLL, DALY, and cost of injury**<br>Variations: PYLL for unintentional injuries; DALY due to unintentional injury; DALY due to unintentional injury for ED visits; DALY due to unintentional injury for hospitalizations; DALY due to unintentional injury for fatalities; Indirect costs and cost per capita due to unintentional injury; Indirect costs due to unintentional injury for ED visits; Indirect costs due to unintentional injury hospitalizations; Indirect costs due to unintentional injury fatalities; Direct costs and cost per capita due to unintentional injury; Direct costs due to unintentional injury for ED visits; Direct costs due to unintentional injury hospitalizations; Direct costs due to unintentional injury fatalities |
| **Poisoning helpline utilization**<br>Variations: Number and rate of poisoning helpline utilization |
| **Self-reported unintentional injuries**<br>Variations: Number, rate and proportion of self reported unintentional injury |
| **Treatment coverage for substance use**<br>Variations: Treatment coverage and proportion for alcohol and drug dependence |
| **Percentage of adult binge drinking**<br>Variations: Number, proportion and rate of adult binge drinking |
| **Communities with access to water safety programs**<br>Variations: Number and proportion of communities with access to water safety education/programs; Number and proportion of communities with children and youth enrolled in "learn to swim" programs in a specific year |
| **Injury prevention legislation and policy 1**<br>Variations: Number of legislations requiring pool fencing; Existence of provincial standard for hot water tap; Existence of window guard bylaws; Existence of provincial law requiring playgrounds meet CSA standards |
| **Injury prevention legislation and policy 2**<br>Variations: Number of homes with working smoke detectors; Number and proportion of homes with tested fire extinguishers; Number and proportion of homes with tested carbon monoxide detectors |
| **Percentage of bicycle helmet use**<br>Variations: Proportion of bicycle helpmet use |
| **Availability of fire and ambulance services**<br>Variations: Number and proportion of fire services responding within certain time |
